# Attractiveness of different esthetic orthodontic wires

**DOI:** 10.1590/2177-6709.25.6.027-032.oar

**Published:** 2020

**Authors:** Deric Meschiari Batista, Melissa Faccini, Fabricio Pinelli Valarelli, Rodrigo Hermont Cançado, Renata Cristina Oliveira, Ricardo Cesar Gobbi de Oliveira, Karina Maria Salvatore Freitas

**Affiliations:** 1 Orthodontic Graduate student. Department of Orthodontics. Ingá University Center Uningá, Maringá.

**Keywords:** Orthodontic wires, Esthetics, Orthodontics

## Abstract

**Objective:**

The aim of this study was to evaluate the attractiveness of different types of esthetic orthodontic wires by laypeople and dentists.

**Methods:**

Five different types of orthodontic wires were evaluated: three esthetic wires (Teflon-coated, epoxy resin-coated and rhodium-coated wires), and two metallic wires (stainless steel and NiTi), as control. Monocrystalline ceramic brackets were installed in the maxillary arch of a patient presenting good dental alignment. The five evaluated wires were attached to the orthodontic appliance with an esthetic silicone elastic and photographed. The photographs were evaluated by 163 individuals, 110 dentists and 53 laypeople. The data were statistically evaluated by two-way ANOVA and one-way ANOVA, followed by Tukey tests.

**Results:**

There was a statistically significant difference in the attractiveness among the wires evaluated; the most esthetic was the rhodium-coated wire, followed by the epoxy resin-coated wire and, finally, the Teflon-coated wire, with no significant difference from the stainless steel and NiTi control archwires. There was no significant difference between the groups of evaluators.

**Conclusion:**

The most attractive was the rhodium-coated wire, followed by the epoxy resin-coated wire and, finally, the least attractive wire was the Teflon-coated wire, without statistically significant difference to the stainless steel and NiTi wires, used as control.

## RESUMO

**Objetivo:**

O objetivo do presente estudo foi avaliar a atratividade de diferentes tipos de fios ortodônticos estéticos, na opinião de leigos e dentistas.

**Métodos:**

Foram avaliados cinco tipos diferentes de fios ortodônticos: três fios estéticos (revestidos com teflon, revestidos com resina epóxi e revestidos com ródio) e dois fios metálicos (aço inoxidável e NiTi), como grupo controle. Braquetes de cerâmica monocristalina foram instalados na arcada superior de uma paciente com bom alinhamento dentário, e os cinco fios avaliados foram fixados ao aparelho ortodôntico com ligadura elástica estética de silicone e fotografados. As fotografias foram avaliadas por 163 indivíduos, sendo 110 dentistas e 53 leigos. Os dados foram avaliados estatisticamente pelos testes ANOVA a um e a dois critérios, seguidos pelo teste de Tukey.

**Resultados:**

Houve diferença estatisticamente significativa na atratividade entre os fios avaliados. O mais estético foi o fio revestido de ródio, seguido pelo fio revestido de resina epóxica e, finalmente, o fio revestido de teflon, sem diferença significativa entre os fios de aço inoxidável e os de NiTi. Não houve diferença significativa entre os grupos de avaliadores.

**Conclusão:**

O mais atraente foi o fio revestido de ródio, seguido pelo fio revestido de resina epóxica e, finalmente, o fio menos atraente foi o fio revestido de teflon, sem diferença estatisticamente significativa para os fios de aço inoxidável e os de NiTi, utilizados como controle.

## INTRODUCTION

Esthetics is the main reason that leads most patients to seek orthodontic treatment.^1^ ^-^ ^4^ The esthetic changes in smile and face achieved through orthodontic treatment have been much approached and conveyed by the media, such as television and internet. The popularization of Orthodontics has generated, in the last years, a significant increase in the number of adult patients seeking for orthodontic treatment.^3^ ^,^ ^5^ ^-^ ^7^ Most of these adult patients and also part of the young patients who intend to start orthodontic treatment have a preference for discreet appliances^8^ and aligners.^9^ This finding shows a current tendency to seek esthetics even during orthodontic treatment, either for social reasons, due to professional requirements or simply for avoiding the “metallic smile”.^8^ ^,^ ^10^ ^,^ ^11^

The major manufacturers of orthodontic appliances began to worry about the esthetics in the mid-1970s. For the manufacture of esthetic orthodontic accessories, the metal that was used in its bases was initially replaced by plastic polymers, which have acceptable clinical properties, even having a much lower stiffness than steel. Subsequently the industry began to use polycrystalline ceramic, acrylic polymers and monocrystalline ceramic, known as sapphire, which is currently the material that provides greater esthetics in orthodontic brackets.^5^ ^,^ ^7^ ^,^ ^8^

To obtain esthetic orthodontic wires, a different problem emerged, compared to orthodontic accessories: the orthodontic wire should keep the metallic mechanical properties. The solution to this problem has been used from the 1970s to the present day, either by painting or coating with esthetic materials the conventional metallic archwires of the most varied alloys, such as stainless steel, titanium-molybdenum and NiTi. The first types of coating to be used were Teflon and epoxy resin.^7^ ^,^ ^8^ ^,^ ^12^ ^-^ ^14^

In an attempt to improve the esthetics of orthodontic wires, some manufacturers have developed the silicone-reinforced nylon-based wires (Optiflex, Ormco, Orange, CA, USA and Optis T, TP Orthodontics, Westville, IN, USA). These wires had an exceptional esthetic, however, they did not have good clinical properties. The manufacturers themselves recommended the use of these wires only on special occasions and for short periods of time. Due to clinical inefficiency, these wires were withdrawn from the market.^14^ Recently, a technique used in the manufacture of jewelry has been used to make esthetic orthodontic wires, the rhodium bath.^13^ ^,^ ^15^

In recent years, esthetic brackets and wires have been widely studied, tested and compared in terms of their coefficient of friction,^16^ surface roughness,^17^ ^,^ ^18^ mechanical properties^17^ and esthetic stability during treatment.^12^ ^,^ ^18^ ^,^ ^19^ Their advantages and disadvantages have already been described in numerous studies in the literature.^7^ ^,^ ^12^ ^-^ ^14^ ^,^ ^17^ Recently, Pinzan-Vercelino et al.^21^ performed a cross-sectional study to evaluate laypersons’ esthetic perceptions of metal archwires with and without esthetic coating and found that the epoxy resin wire was the most esthetic. But no study compared the attractiveness of several types of esthetic wires evaluated by both laypeople and dentists.

In this context, the present study aimed at evaluating the attractiveness of the different types of esthetic orthodontic wires by laypeople and dentists.

## MATERIAL AND METHODS

In this study, five types of 0.016-in orthodontic wires were inserted into a single esthetic orthodontic appliance at different times and photographed in the smile.

Three esthetic wires were evaluated and two metallic wires were used as controls, as follows:

1. Epoxy resin-coated wire (Ever White NITI, American Orthodontics, Sheboygan, Wisconsin, USA).
2. Teflon-coated wire (Spectra, Dentsply GAC, Islandia, New York, USA).
3. Rhodium-coated wire (Sentalloy High Aesthetic, Dentsply GAC, Islandia, New York, USA).
4. NiTi wire (Flexy-NiTi Thermal, Orthometric, Marília/SP, Brasil).
5. Stainless steel wire (Morelli, Sorocaba/SP, Brasil).

In the orthodontic clinic of one of the authors, a female patient with great smile esthetics, good teeth alignment and leveling and normal overjet and overbite was selected. The patient signed a free and informed consent form accepting the participation and permitting the use of the photographs for research and academic purposes.

Monocrystalline ceramic brackets (Inspire Ice, Ormco, Orange, California, USA) were bonded from the right to the left second maxillary premolars. No appliance was bonded in the mandibular arch.

**Figure 1:**
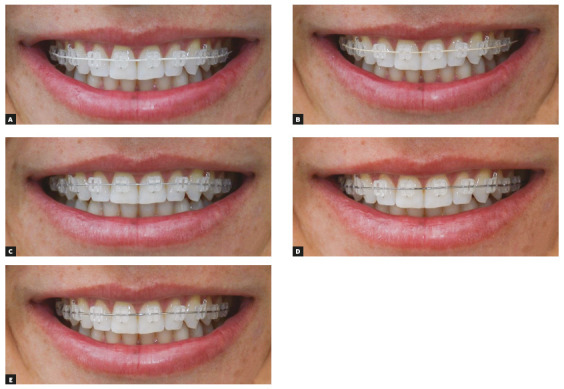
Photographs of the evaluated wires: A) Ever White NiTi (epoxy resin), B) Spectra (Teflon), C) Sentalloy High Aesthetic (rhodium), D) Flexy NiTi Thermal, E) stainless steel.

After appliance installation, each wire was inserted and attached to the brackets with silicone ligatures (SiLi-Tie Clear, Dentsply GAC, Iceland, New York, USA). Photographs were taken on artificial light without flash, with a D3200 camera with Nikkor 18/140-DX lens (Nikon LTDA, Minato, Tokyo, Japan), in frontal norm, with the patient in posed smile. The camera was attached to a tripod in front of the dental chair and all photographs were taken in the same position when the chair’s backrest was in the most possible vertical position. All images were standardized using Adobe Photoshop software (CC 2016 version, Adobe Systems, San Jose, California, USA), with 300 dpi and with the same zoom (Fig. 1).

**Figure 2:**
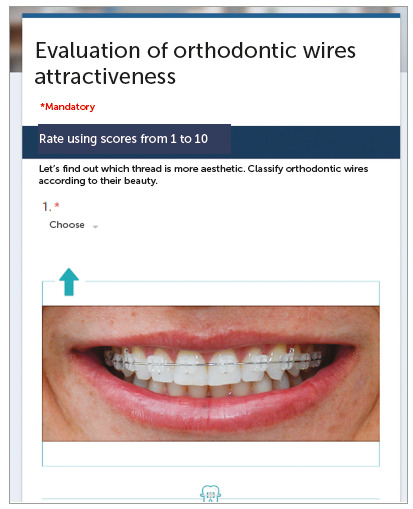
Screen capture of the Google forms site, where raters evaluated the wires.

The five images (one for each evaluated wire) were used to compose a digital album, using a Google form (Fig. 2), and were evaluated by the raters. The images were presented in a random order, one underneath the other, for evaluation. 

The study participants evaluated each wire individually and blindly, and rated from 1 to 10, based on the attractiveness - 1 being the least attractive and 10 being the most attractive wire. The evaluators could look and examine each of the images and compare them as they wanted for 10 minutes in total.

The link to evaluate the attractiveness of the different esthetic orthodontic wires was sent by e-mail and by WhatsApp message to laypeople and dentists, selected from the *Centro Universitário Ingá Uningá* (Brazil) university’s database of former students. Inclusion criteria were: Dentists graduated for at least 5 years; laypeople graduated in another area than Dentistry, also for at least 5 years; age from 20 to 40 years. The dentists’ specialty was not considered as a criterion.

About 150 messages and e-mails were sent for each group of evaluators, 300 in total, from which 163 individuals answered, resulting in a response rate of 54.3%. The response rate was 73.3% for dentists and 35.3% for laypeople. The laypeople responded less to the messages than the dentists, perhaps because they were less interested in the subject of the questionnaire.

The total number of evaluators was 163 individuals, 110 dentists (49 males, 61 females) and 53 laypeople (22 males, 31 females). The mean age was 26.78 for the whole sample, 25.31 for the laypeople and 28.72 years for the dentists.

## STATISTICAL ANALYSIS

The two-way analysis of variance test was performed considering the types of wire and evaluators. Since there was statistically difference in the types of wire, the one-way ANOVA and Tukey tests were performed.

Descriptive statistics were also performed for each evaluated wire and each group of raters.

The tests were performed with Statistica software (Statistica for Windows version 7.0, Statsoft, Tulsa, Oklahoma, USA). Results were considered significant for *p*< 0.05.

## RESULTS

The results of the two-way analysis of variance showed statistically significant difference in the interaction and among the different types of evaluated wires, and no significant difference between the evaluators, dentists or laypeople (Table 1).

**Table 1:** Results of the evaluation of the type of wire and evaluators (two-way analysis of variance).

Type of variation	DF	F	P
Interaction	4	4.229	0.002*
Type of wire	4	28.312	0.000*
Type of evaluator	1	2.930	0.087
Intercept	1	4889.689	0.000*
Error	968		

* Statistically significant for *p*< 0.05. DF = degree of freedom, F = Variance.

The rhodium wire showed to be the most attractive with a significant difference for all the other wires, followed by the epoxy resin-coated wire, which also showed significant differences to the other wires and, finally, the Teflon-coated wire, which presented similar attractiveness to the control stainless steel and NiTi wires (Table 2).

**Table 2:** Results of the comparison of the attractiveness among the wires (one-way ANOVA and Tukey tests) (n=163).

Variable	Epoxy resin	Teflon	Rhodium	Stainless steel	NiTi	p
	Mean (SD)	Mean (SD)	Mean (SD)	Mean (SD)	Mean (SD)	
Attractiveness	6.11 (2.35)^A^	4.90 (2.38)^B^	7.42 (2.22)^C^	5.28 (2.45)^B^	4.76 (2.21)^B^	0.000*

* Statistically significant for *p*< 0.05. Different letters in a row indicate the presence of a statistically significant difference.

Table 3 shows the descriptive statistics of each evaluated wire and each group of raters.

**Table 3:** Means and standard deviations of the attractiveness of each wire evaluated for each group of raters.

WIRES	DENTISTS (n=110)		LAYPEOPLE (n=53)	
	Mean	SD	Mean	SD
Stainless steel	5.04	2.17	5.76	2.75
NiTi	4.50	2.05	5.30	2.46
Epoxy resin	6.10	2.16	6.13	2.66
Rhodium	7.54	2.01	7.16	2.62
Teflon	4.91	2.39	4.90	2.40

## DISCUSSION

The objective of this work was to evaluate the attractiveness of the different types of esthetic orthodontic wires. In order to reduce the interference in the evaluators perception, monocrystalline ceramic accessories and silicone esthetic ligatures were used, since these materials were the most esthetic of their categories.^8^ ^,^ ^21^ Besides, a patient with a great occlusion and smile esthetics, good teeth alignment and leveling and normal overbite and overjet was selected for orthodontic appliance bonding, in order to not interfere in the evaluation of the wire esthetics. 

The rate of wire attractiveness did not present statistically significant differences between the evaluators (Table 1). This way, it can be assumed that in the evaluation of the attractiveness of orthodontic wires, a specialist’s view did not differ from the view of a layperson. 

The Teflon-coated wire and the stainless steel and NiTi wires presented similar attractiveness and were the least attractive evaluated wires (Table 2). The NiTi and stainless steel wires are metallic and uncoated, so they were considered as control for a reference parameter in the evaluation of the other wires that have different types of coating. The Teflon-coated wire was expected to be more attractive than the conventional metallic wires, and although it was whitish in color and marketed as esthetic, it did not differ from the metallic non-esthetic wires. It was one of the first wires sold and marketed as esthetic^14^ and maybe this is the reason for the worst esthetics when compared to the epoxy resin and rhodium-coated wires, since these wires were developed later.

The epoxy resin-coated wire showed higher attractiveness than the NiTi, Teflon-coated and stainless steel wires, and lower attractiveness than the rhodium-coated wire (Table 2). For being considered esthetic, this result was expected, compared to the conventional metallic wires. Pinzan-Vercelino et al.^21^ found in their study that the epoxy resin wire was the most attractive. However, in their study, the wires were evaluated only for 30 seconds each. In the present study, the evaluators had 10 minutes to evaluate all the wires, so they were able to compare the wires together. Probably when compared, the white coating appeared less attractive, justifying the differences with the Pinzan-Vercelino’s study.^21^

The white coating of the epoxy resin appears to be more attractive than the Teflon yellowish coating and the conventional metallic wires. However, the epoxy resin wires, when in contact with the oral environment resemble to those of Teflon, and could undergo corrosion, drastic alterations in color, besides peeling in some parts, due to masticatory and friction forces, allowing the metal to be revealed, which causes discomfort to the patient and is unesthetic.^12^ ^-^ ^14^

The rhodium-coated wire showed the highest esthetic attractiveness among all the evaluated wires, followed by the epoxy resin-coated wire, with a statistically significant but numerically small difference (Table 2). The interesting about this result is that the most esthetic wire is not necessarily the whitest, since the rhodium bath gives a silver color, with a very clear shade, to the orthodontic wire, and may better mimic the shade of the teeth than the white color. This result is understandable since rhodium-coated wire has been developed more recently, using a more modern technique, applied in jewelry production.^13^ ^,^ ^15^ In addition to the fact that rhodium-coated wire has presented the greatest attractiveness, it also presents considerable clinical advantages, being the esthetic wire that presents less color alteration and less corrosion of the esthetic coatings available in the market.^22^ ^,^ ^23^

Esthetic brackets and wires should not only be attractive, but also efficient in orthodontic movement. It is important that the accessories and wires meet the patient’s esthetic expectations with clinical performance expected by the professional.^13^ The wire with the best clinical performance in previous studies^13^ ^,^ ^22^ ^,^ ^23^ was also considered the most attractive in the present study.

However, despite being the best evaluated wire regarding attractiveness, the scores given to the rhodium-coated wire in the present study (mean score of 7.42) are far from excellent, which shows that there is still a need for improvement. The companies can still work on new technologies to improve the esthetic wires, also seeking the best mechanical properties for orthodontic movement.

## CONCLUSION

The most attractive wire was the rhodium-coated, followed by the epoxy resin-coated wire. The Teflon-coated wire was the least attractive, without significant difference from the control metallic wires. 
